# Power-efficient low-temperature woven coiled fibre actuator for wearable applications

**DOI:** 10.1038/srep36358

**Published:** 2016-11-04

**Authors:** Maki Hiraoka, Kunihiko Nakamura, Hidekazu Arase, Katsuhiko Asai, Yuriko Kaneko, Stephen W. John, Kenji Tagashira, Atsushi Omote

**Affiliations:** 1 Panasonic Corporation, Advanced Research Division 3-4 Hikaridai, Seika-cho, Kyoto, 619-0237, Japan

## Abstract

A fibre actuator that generates a large strain with high specific power represents a promising strategy to develop novel wearable devices and robotics. We propose a new coiled-fibre actuator based on highly drawn, hard linear low-density polyethylene (LLDPE) fibres. Driven by resistance heating, the actuator can be operated at temperatures as low as 60 °C and uses only 20% of the power consumed by previously coiled fibre actuators when generating 20 MPa of stress at 10% strain. In this temperature range, 1600 W kg^−1^ of specific work (8 times that of a skeletal muscle) at 69 MPa of tensile stress (230 times that of a skeletal muscle) with a work efficiency of 2% is achieved. The actuator generates strain as high as 23% at 90 °C. Given the low driving temperature, the actuator can be combined with common fabrics or stretchable conductive elastomers without thermal degradation, allowing for easy use in wearable systems. Nanostructural analysis implies that the lamellar crystals in drawn LLDPE fibres are weakly bridged with each other, which allows for easy deformation into compact helical shapes via twisting and the generation of large strain with high work efficiency.

Fibre actuators are anticipated to add a new mechanical function to flexible electronics for wearable garments or biomimetic robotics^1^,^2^,^3^,^4^. A coiled fibre actuator composed of a twisted hard crystalline polymer or carbon nanotube fibres has attractive features, such as being strong, lightweight, fast, scalable, washable, and weavable into various threads or fabrics^5^,^6^,^7^,^8^. However, such actuators are driven by the thermal strain of fibres heated to more than 100 °C, which causes thermal degradation of peripheral flexible components. The high driving temperature is a consequence of the glass-transition temperature *T*_g_ exceeding 100 °C in the case of hard crystalline polymers because little thermal strain is induced as the samples are heated to temperatures approaching their *T*_g_.

The large anisotropic thermal strain of a drawn crystalline polymer originates from stretched tie-molecules, which cause large entropy elasticity along the drawn axis^9^. The tie-molecules are included in non-crystalline parts of the crystalline polymer. In general, crystalline polymers become hard when drawn into fibres because of realignment of the fibril configuration. Especially large entropy elasticity of highly stretched tie-molecules causes negative linear thermal expansion (NLTE) of the fibres^9^. When highly drawn crystalline polymer fibres are twisted and coiled, their anisotropic strain is converted into a large linear displacement ∆*L* of the coil length, which is approximately modelled by the following approximation:


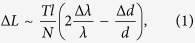


where *T*, *l*, *n*, *N*, *λ*, and *d* denote the total number of twists applied on coiled fibre, the length of the twisted fibre, the number of twists before coiling, the number of coils, the fibre length before twisting, and the fibre diameter, respectively^5^.

To our knowledge, the literature contains no reports of the fabrication of a drawn crystalline polymer fibre suitable for coiled actuators. We consider one of the ideal structural models to be analogous to a series of sarcomere structures comprising skeletal muscle fibres^10^. This structure consists of actuating parts periodically bridged between hard lamellar boundaries that confine the strain of the actuating fibrils in the muscle fibre axis. Similarly, fibrils of drawn crystalline fibres that consist of tie-molecules periodically bridged between hard lamellar boundaries along the fibre axis could generate large NLTE (Fig. 1a).

To investigate how fibril morphology effectively functions to enable a coiled actuator to operate at low temperatures, we selected polyethylene, which has the simplest molecular structure but exhibits various morphologies and mechanical properties. Polyethylene is classified on the basis of its molecular weight (MW 5 × 10^2^ to 6 × 10^6^ g mol^−1^) and density (0.86–1.0 g cm^−3^), which are governed by the branch structure of the polymer chain^11^. It exhibits a low *T*_g_ between −40 °C and −140 °C, which implies that the entropy elasticity of tie-molecules is activated at room temperature.

In general, hard fibres are obtained from high-density polyethylene (HDPE); numerous attempts to increase its hardness for industrial applications have been reported^11^. However, its crystallinity is greater than 70%, which reduces the NLTE because of the presence of fewer tie-molecules.

To exploit the NLTE of a crystalline polymer, we focused on using linear low-density polyethylene (LLDPE; we used LLDPE with 0.92 g cm^−3^, *T*_g_ = −110 °C, MW ≈ 10^5^ g mol^−1^). This LLDPE consists of short branched polyethylene chains typically synthesized from ~2 mol % of *n*-butylene mixed with ethylene, and is transformed into hard fibres via hot drawing; fibres drawn from soft fibres (*E* ≈ 0.2 GPa) exhibit a Young’s modulus *E* greater than 2 GPa^11^,^12^. Because of its short branched chains, crystallization of fibrils is confined to approximately 60%; therefore, tie-molecules remain in the non-crystalline parts despite the high drawing ratio of the fibres^12^,^13^,^14^.

Although LLDPE is widely used in industry, to our knowledge, in the case of fibres sold as soft elastic filaments, hard LLDPE drawn fibres have not been used in any application^11^,^15^. Furthermore, the thermal expansion behaviour of hard LLDPE drawn fibres has not been reported. In this article, we extrude and draw crystalline polymer fibres suitable for use in a coiled actuator and propose an efficient coiled actuator based on highly drawn LLDPE; the proposed coiled actuator exhibits high power and requires a small heat input compared to other reported coiled actuators.

## Results

### Fabrication of drawn and coiled fibres that exhibit large thermal contraction

**Figure 1:**
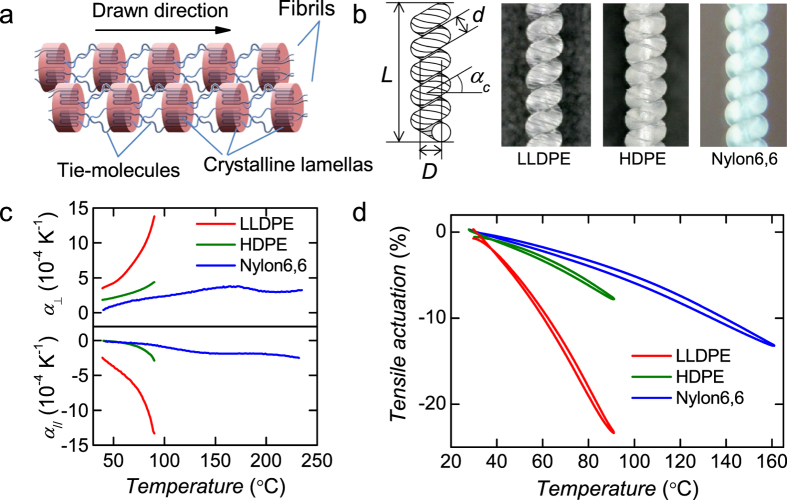
Drawn fibres and coiled actuators fabricated from crystalline polymers. (**a**) Ideal structural model of repeating units of oriented tie-molecules and crystalline lamellas along the drawn direction. (**b**) (left) Schematic of a twisted coil; (right) optical microscope images of coiled fibres. (**c**) Thermal expansion coefficient of drawn fibres along the polar direction (*α*_⊥_) and along the fibre axis (α_//_), as measured by TMA. d) TMA results for coiled fibres.

We extruded and drew hard LLDPE fibres (*E* = 5.5 GPa at drawing ratio *λ*/*λ*_0_ = 10, where *λ*_0_ is the fibre length before drawing). For comparison, we also fabricated hard drawn fibres with similar stiffness to LLDPE from HDPE (0.96 g cm^−3^, *E* = 5.7 GPa, *λ*/*λ*_0_ = 24) and nylon6, 6 (1.14 g cm^−3^, *E* = 6.6 GPa, *λ*/*λ*_0_ = 5). We then twisted the fibres until they were fully coiled. Figure 1b shows a schematic and microscope images of the coiled fibres. Generally, displacement of the coiled actuator increases with increasing spring index *c* = *D*/*d*, where *D* and *d* denote the mean diameter of the coil and the diameter of the fibre, respectively. The coiled LLDPE fibre is compact, with *c* = 0.7 and a coil bias angle *α*_c_ = 32°, as calculated from the microscope image; for comparison, the *c* and *α*_*c*_ of the coiled HDPE fibre are 1.1 and 20°, respectively, and those of the coiled nylon6, 6 fibre are 1.2 and 15°, respectively. Interestingly, although the tested drawn LLDPE was a hard filament, the coiling-process deformed circularity of the fibre diameter increased to 200% from 104%. By contrast, the diameters of HDPE and nylon6, 6 did not deform from a circle until they were broken by shear stress.

Figure 1c shows the temperature dependence of the thermal expansion coefficients of the drawn fibres. In contrast to drawn HDPE and nylon6, 6 fibres^9^,^11^, the drawn LLDPE fibre exhibited a large negative *α*_//_ and positive *α*_⊥_, on the order of ±10^−3^ K^−1^ for *λ*/*λ*_0_ > 5 (Fig. S1). Figure 1d shows thermomechanical analysis (TMA) results for coils under 20 MPa of tensile stress. We denote a load applied on a cross section of a fibre diameter as the tensile stress applied on the coiled fibre. Because of the large anisotropic thermal expansion of LLDPE, the displacement of the LLDPE was larger than that of the other polymers despite its small *c*.

We estimated the actuation using equation (1). The thermal expansion of untwisted fibres, the parameters used for calculation, and results are summarized in Fig. S2 and table S1. The difference between calculated and measured motion was relatively small in several ten percent under smaller actuation below 10%. Although the error increased for larger actuation as the thermal change of *T* and elastic modulus of the coil remained unmodeled, equation (1) is convenient to estimate the performance of the coiled actuator.

In such actuations, the work efficiency *η* of coiled LLDPE, HDPE, and nylon6, 6 actuators between 30 °C and 90 °C are estimated to be 1.2%, 0.3%, and 0.2%, respectively. The *η* will be discussed later with an analytical model.

**Figure 2:**
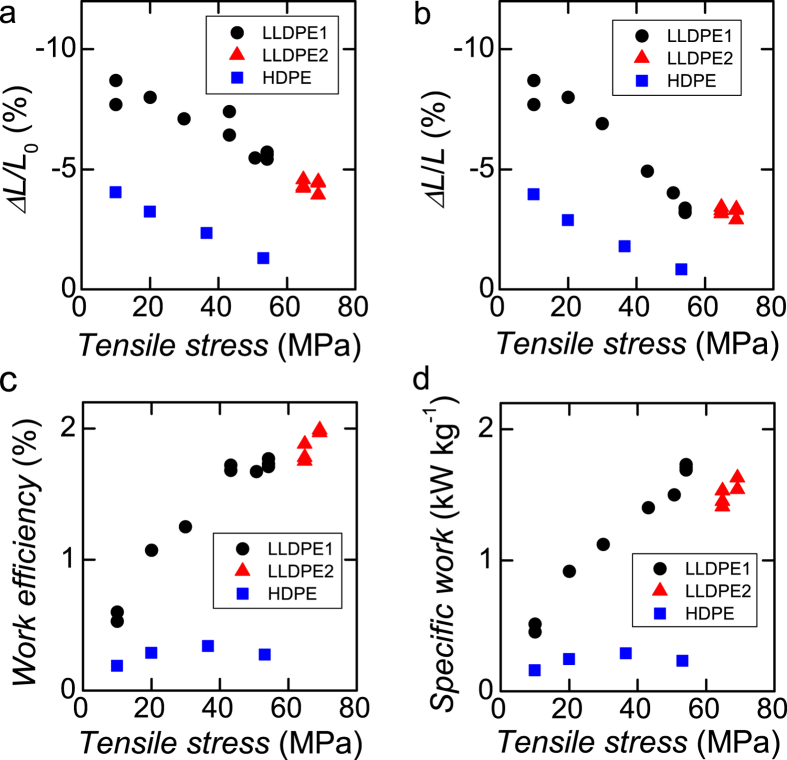
Actuation of coiled LLDPE and HDPE fibre actuators under various tensile stress. The black closed circles and red closed triangles are coiled LLDPE fibres fabricated by twisting fibres of ϕ170um diameter under tensile stresses of 20 MPa (LLDPE 1, *c* = 0.7) and 30 MPa (LLDPE 2, *c* = 0.5), respectively. The blue closed squares are coiled HDPE fibres fabricated by twisting fibres with ϕ180um diameter under tensile stresses of 20 MPa (LLDPE 1, *c* = 1.1). The HDPE fibres were broken by twisting under tensile stresses of 30 MPa. All actuators in these figures were driven for 0.65–1.1 sec. (**a**) Displacement of the actuators normalized by actuator length under tensile stress. (**b**) Displacement of the actuators normalized by natural length of the actuator. (**c**) Work efficiency estimated from the work shown in (**b**) divided by the heat change of the actuator. (**d**) Specific work of the actuators. Plots are shown only for actuators driven for 0.65–0.7 sec.

Figure 2 shows the actuation of LLDPE coil actuated at low temperatures and heated to 55 °C from room temperature (25 °C) under various tensile stresses. The actuation of HDPE coils is also shown for comparison. The LLDPE coils with *c* = 0.7 and exposed to 54 MPa tensile strength broke down when heated. By contrast, the LLDPE coils with *c* = 0.5 held a large tensile stress of at least 69 MPa. Thermal contraction under heavy load at 69 MPa, as shown in Fig. 2a, decreases to approximately 40% of the strain under 10 MPa of tensile stress. Figure 2b shows thermal contraction normalized by the natural spring length. The compact LLDPE coil with *c* = 0.5 exhibits a smaller difference because its spring constant is greater than that of the LLDPE coil with *c* = 0.7.

Figure 2c,d shows the *η* and the specific work, respectively, under various loads. The largest *η* (2%) was obtained at 69 MPa, with a work output of 1600 W kg^−1^. This performance is the best reported thus far for a thermally driven fibre actuator, including those composed of shape memory alloys^5^. A maximum specific work of 1700 W kg^−1^ was obtained at 54 MPa for the LLDPE coil with *c* = 0.7.

In our result, the *η* of the HDPE coils was rather smaller than that of the LLDPE coils and a ultra high-molecular-weight polyethylene (UHMWPE) coil reported elsewhere^5^. The UHMWPE fibre consists of high crystallinity more than 90%, and is an extraordinary stiff fibre with Young’s modulus of 70 GPa. Even though *η = *1.3% was reported on the hard UHMWPE coil by applying 10 msec pulse of Joule heat under 230 MPa of the tensile stress, our hard HDPE fibres just hold tensile stress below 40 MPa.

### Nanostructural analysis

**Figure 3:**
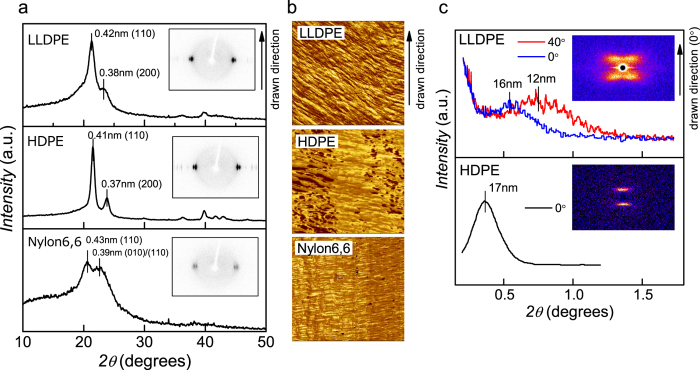
Nanostructure analysis of drawn fibres. (**a**) WAXD peak profiles taken from the equatorial line of the inset WAXD images. (**b**) AFM phase images (1 μm × 1 μm) of cross-sectional surface of the drawn fibres cut along the fibre axis. The dark and light areas correspond to harder and softer parts of the fibrils, respectively. (**c**) Peak profiles of SAXS of the drawn LLDPE and HDPE lines. The angle of the peak profile is defined according to the drawn direction. Insets show SAXS images.

We investigated the nanostructures of drawn fibres to discuss the mechanism of large displacement and high work efficiency of coiled fibre actuators. Wide-angle X-ray diffraction (WAXD) patterns of all of the samples indicate that the *c*-axis of the crystalline parts aligns with the drawn axis (Fig. 3a)^14^,^16^,^17^. In contrast to the similar WAXD results for all of the fibres, atomic force microscopy (AFM) images of the cross sections of drawn fibres (Fig. 3b) and small-angle X-ray scattering (SAXS) patterns (Fig. 3c) indicate that the long-range order differed substantially among the fibre samples. In the AFM images, the drawn LLDPE fibre consists of stacked fine linear patterns with a width of 10–20 nm. By contrast, the drawn HDPE fibre consists of larger domains of approximately 20–100 nm. The fine linear patterns of LLDPE are consistent with the SAXS pattern, which shows long-range order of 12 nm to 16 nm distributed at an angle of approximately 40° from the fibre axis. The long-range ordering distance is consistent with the lamellar structure of LLDPE^13^,^18^. We conclude that the fine patterns are crystalline lamellas periodically interleaved by non-crystalline components along the fibre direction. By contrast, the SAXS pattern of HDPE shows highly ordered long-range order at 17 nm, which is smaller than the domain structures observed by AFM; therefore, crystalline lamellas are connected and aligned with each other.

### Actuator threads and their performance

**Figure 4:**
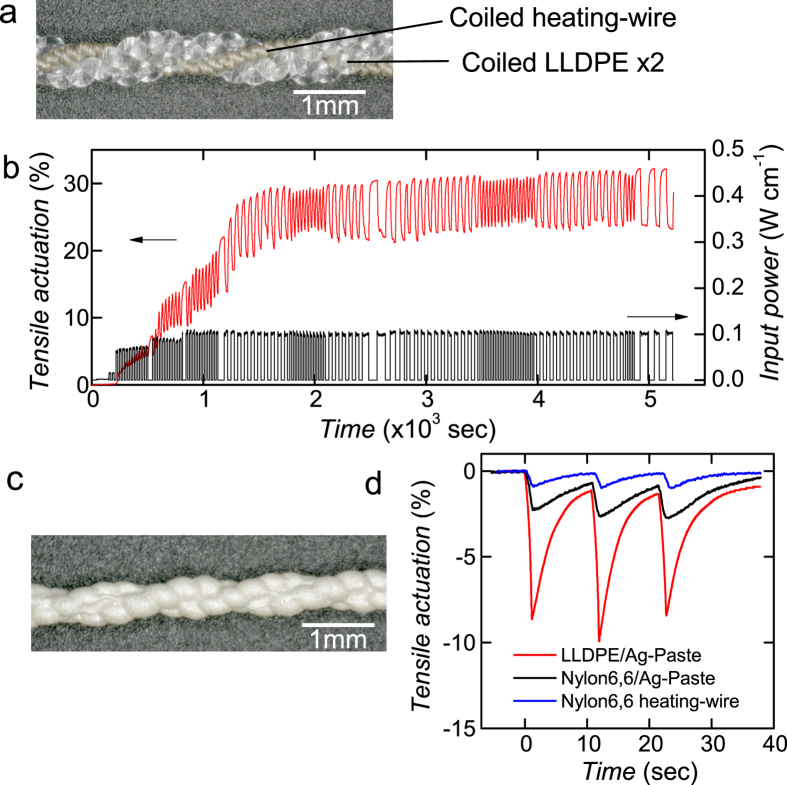
Coiled LLDPE actuators composed of coiled LLDPE and an electrical resistive heater. (**a**) Photographs of 3-ply yarn composed of a couple of coiled LLDPE fibres with ϕ0.2 mm diameter and heater wire with 100 denier (LLDPE/heating-wire). (**b**) Actuation of the LLDPE/heating-wire using 110 pulses of input power, including initialization steps of 30 pulses (up to 1500 sec). (**c**) Photographs of the 2-ply yarn of coiled LLDPE fibres with ϕ0.2 diameter coated with a conductive elastomer (LLDPE/Ag-paste). (**d**) Actuation of LLDPE/Ag-paste by electrical resistive heating with an input power of 0.14 W cm^−1^ for 1 sec. 2-Ply yarns of conductive elastomer-coated coiled nylon6, 6 fibres (nylon6, 6/Ag-paste, *c* = 1.2) and Ag-plated coiled nylon6, 6 multifilament with 300 denier (nylon6, 6 heating-wire) were also fabricated and measured for comparison. All actuators have almost same cross sectional area of 0.03 mm^2^. The nylon6, 6 heating-wire of 100 denier was also used as heater wire shown in (**a**). As the nylon6, 6 heating-wire exhibited very small actuation, a contribution of the actuation on the LLDPE/heating-wire is negligible.

As the simplest method for fabricating an electrically driven coiled LLDPE actuator, we wound coiled LLDPE fibres with a heating-wire (LLDPE/heating-wire, Fig. 4a. Figure 4b shows the cyclic actuation of a freshly prepared LLDPE/heating-wire under 10 MPa of tensile stress. The LLDPE/heating-wire actuator was operated under an input power of 0.1 W cm^−1^, consisting of a series of pulsed voltages with durations of 15, 30, or 70 sec. After the initial 30 cycles, with irreversible extension of approximately 30%, the cyclic actuation became stable. This initialization was necessary only at the first actuation, and would stabilize the slack of coiled LLDPE fibres in 2-ply yarn.

The similar amount of an initial irreversible-extension was also observed in an initial heat cycle of the TMA. The TMA of coiled HDPE and nylon6, 6 fibre caused irreversible extension of approximately 20%. The smaller irreversible extension of coiled HDPE and nylon6,6 fibre compared to coiled LLDPE fibre is believed to be due to the well linkage of the lamella crystals connected by the crystalline bridges.

Displacement was almost saturated at approximately 8% for a duration of input power of 3 sec with *η* = 0.08%, which would be caused by large thermal dissipation from the heating-wire to air and could be improved by designing a weaving arrangement comprising LLDPE and heating-wire mixed yarn.

To achieve rapid actuation by improving heat transfer from the heater to the coiled fibre, we coated the coiled LLDPE fibre with a conductive elastomer (LLDPE/Ag-paste, Fig. 4b). For comparison, we fabricated a coiled nylon6, 6 fibre coated with a conductive elastomer (nylon6, 6/Ag-paste) and a coiled Ag-plated nylon6, 6 multifilament (nylon6, 6 heating-wire). All actuators have nearly identical cross-sectional areas of 0.03 mm^2^, comprised of LLDPE or nylon6,6 monofilaments with ϕ0.2 mm diameter and the nylon6, 6 multifilament with 300 denier. When we applied an input power of 0.16 ± 0.03 W cm^−1^ (190 ± 30 W g^−1^) to the conductive elastomer of LLDPE/Ag-paste under a tensile stress of 10 MPa, it contracted by −10 ± 2% sec^−1^ for the three samples, whereas the nylon6, 6/Ag-paste contracted by only −3.3% sec^−1^ at 200 W g^−1^ (Fig. 4d, see also movie S1 in the Supplementary information). The work efficiency *η* of the LLDPE/Ag-paste and nylon6, 6 during contraction were 0.31% and 0.06%, respectively. Due to thermal dissipation of the input heat into air, the *η* reduced into half of the result shown in Fig. 2c under 10MPa tensile stress (0.6%). Although *η* varies with device structure and input power, the coiled LLDPE fibre may reduce the energy consumption to approximately 20% of that reported for coiled crystalline polymer fibres in ref. 5.

**Figure 5:**
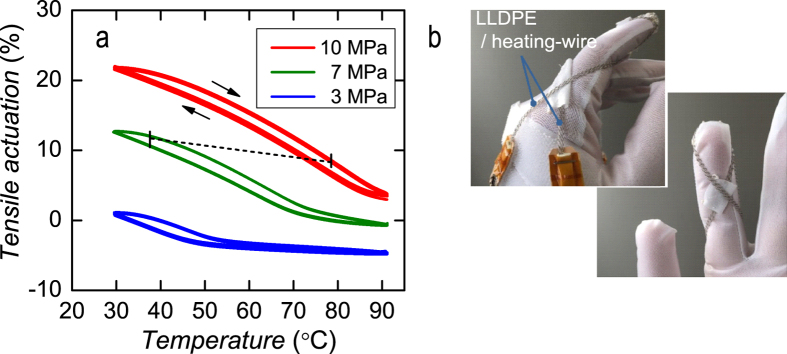
Coiled LLDPE actuators composed of electrical resistive heater. (**a**) Actuation of LLDPE/heating-wire under various loads. A diameter of the fibres are same as in Fig. 4a,b. (**b**) Photographs of actuators attached to a fabric glove taken from (left) the back side and (right) palm side. Six LLDPE/heating-wires are attached to the glove. The dashed line in (**a**) indicates movement when the actuator is bent 6 mm from the extended position with a finger.

Because of its low operating temperature, the coiled LLDPE actuator can be attached to general fabric. Figure 5a shows the TMA results for LLDPE/heating-wire under various loads. The curves show contour lines at altitudes of the tensile strains plotted in the strain-temperature plane; these contour lines are used for mechanical design. The dashed line in Fig. 5a indicates a trace of actuation estimated from a measurement of the stress–strain change when six 100-mm-long braids of LLDPE/heating-wire are attached between the end of the index finger and back of a fabric glove (Fig. 5b) to bend the index finger approximately 6 mm from the extended position. By applying 50 mA for each LLDPE/Heating-wire, we confirmed that the finger pulling the glove was bended 3 mm by electrical actuation, which was only half of the expected performance. The heat dissipation from the heating-wire raises the temperature of the finger skin of the inner glove to 40 °C, of which thermal loss would reduce the saturated strain of the LLDPE/heating-wire.

## Discussion

As shown in Fig. 1c, large anisotropic thermal expansions are the main factor affecting *η*. We now consider how other factors, such as coil shape, affect *η*. By taking the model describing the geometrical change of a coiled fibre surface during heating operation, we calculate *η* as the work output divided by the energy input:


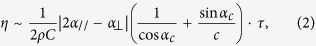


where *ρ* is density of the fibre, *C* is the specific heat, and *τ* is the shear stress. Derivation of this formula is described in Supplementary information. We also summarized the estimation and experimental results of *η* in table S2. The estimation of the *η* almost agreed with the experimental results within an error of about 20 percent except for the actuation of the LLDPE coil under heavy load (69 MPa tensile stress). The difference of the *η* between the estimation and the experimental result under heavy load is due to the unmodeled effect of load on *α*_//_ and _*σ*⊥_.

According to equation (2), one of strategy for high work efficiency is to use very stiff fibre showing large breakdown tensile stress such as UHMWPE fibre as demonstrated in ref. 5. The LLDPE fibre produces alternative solution for large actuation under low temperature range, as equation (2) suggests that a compact shape of the coil with large *α*_c_ and small *c* contributes to a high work efficiency. This model implies that a general approach to improve the thermal response just by increasing *c* (and also by making *α*_c_ smaller) results in worse work efficiency because of more input energy is required. On the basis of the nanostructural analysis results (Fig. 3), we propose that the differences between the LLDPE fibres and other fibres is attributable to the strength of inter-fibril connections of adjacent crystalline lamellas (see schematic model in Fig. 1a). Entropy elasticity of the stretched tie-molecules in the drawn direction in the LLDPE fibrils is barely blocked by weak crystalline bridges between adjacent lamellas, in contrast to HDPE, whose stiffness arises from the connected rigid crystalline network, which should block entropy elasticity. Entropy elasticity also contributes to the LLDPE coil assuming the compact helical bulk structure. This compact shape has the advantage of increasing the density of coiled fibres when the fibres are made into a bundle. The coiled LLDPE fibres would also be suitable for producing a high power actuator thread used for sewing, given the relative smoothness of the coiled fibre surface compared to conventional coiled fibre actuator threads.

In conclusion, we presented a new coiled actuator fabricated from drawn LLDPE fibres; the fabricated actuator functions under low operating temperatures. Our actuator generates strain as high as 23% when heated from 30 °C to 90 °C despite the highly compact shape of the coil (*c* < 1), which leads to efficient actuation with small heat input. A high work efficiency of 2% is attained with a large specific power of 1600 W kg^−1^ under a tensile stress of 69 MPa when the actuator is heated to 55 °C. We demonstrated the actuator being driven by electrical resistive heating. It was operated with only 20% of the energy consumption of conventional actuators based on nylon6, 6 or hard polyethylene. Because of its low operating temperatures, the actuator can be combined with common fabrics or stretchable conductive elastomers without thermal degradation, which enables the use of fibre actuators with a large output power for novel wearable applications. The performance of the actuator in a power-assisted glove will be improved by thermal insulation of the fabric glove and through more sophisticated mechanical design of the textile of a glove functionalized as a soft exoskeleton^19^.

## Methods

### Fabrication of drawn crystalline fibres and coils

We purchased LLDPE (0.92 g mL^−1^), HDPE (0.96 g mL^−1^), and nylon6, 6 (1.14 g mL^−1^) in pellet form from Sigma-Aldrich (Tokyo, Japan). We fabricated the 10-times-drawn fibre of LLDPE according to the following procedure: first, the pellets of LLDPE were dried in a vacuum oven at 90 °C at 0.1 bar for 2 h. The pellets were then extruded by a melt extruder (IMC-1716, Imoto Machinery Co. Ltd.) at 160 °C at 0.79 mL min^−1^ from a ϕ1 mm opening of an extruder-die and rolled onto a bobbin at 2 m min^−1^. A cooled LLDPE fibre with ϕ0.6 mm diameter was obtained. Then, to apply the drawing ratio of 10, we extended the LLDPE fibre on the heating plate of a drawing machine (IMC-60B3, Imoto Machinery Co. Ltd.) at 80 °C; the feeding and winding rollers of the drawing machine operate at 0.4 m min^−1^ and 4 m min^−1^, respectively. Finally, we obtained a drawn LLDPE fibre with a ϕ0.2 mm diameter. We fabricated a 10-times-drawn fibre of LLDPE with a ϕ0.12 mm and ϕ0.17 mm using the same procedure, but the fibre was extruded by the melt extruder at 0.31 mL min^−1^ and rolled onto a bobbin at 2.2 m min^−1^ and 1.1 m min^−1^, respectively. We fabricated a 24-times-drawn HDPE fibre with a ϕ0.18 mm diameter using the same procedure, but the feeding and winding rate of the drawing machine were 0.4 m min^−1^ and 10 m min^−1^, respectively, and the temperatures of the heating plate was 100 °C. We fabricated a 5-times-drawn fibre of nylon6, 6 with a ϕ0.13 mm and ϕ0.2 mm diameter using the same procedure but with the following parameters: before extrusion, the sample was dried in an oven at 210 °C for 6 h at 0.1 bar, then extruded at 270 °C and drawn using a feeding roller at 0.8 m min^−1^ and a winding roller at 4 m min^−1^ to apply a drawing ratio of 5.

Each drawn fibre was twisted into a coil using a home-made twisting machine, which can apply definite tension and control position via a closed-loop feedback system that consists of a servomotor and tension sensor. The number of twists and speed (440 rpm) were also controlled.

### Characterization of drawn fibres and coils

We measured the Young’s modulus E using a Shimadzu EZ-LX. The temperature dependence of the fibre strain under constant tensile stress was measured via thermomechanical analysis (TMA) (Thermo Plus TMA8310, Rigaku Corporation) for a sample length L = 10 mm. The ramp/cool rate of the electric furnace was ±2 K min^−1^. In Fig. 1c, we also used TMA to measure the thermal expansion coefficient of the fibre diameter. A fibre was positioned beneath the sample probe of the TMA and a constant force of 98 mN was applied; this force was sufficiently small to provide a general crystalline polymer fibre of ϕ0.1 mm diameter and with E > 1 GPa. An error of reference of approximately 5.5 ± 1.0 nm K^−1^, which is less than 10% of the strain of the fibre diameter, was compensated for by subtraction from the results. We estimated the ideal work efficiency η under adiabatic conditions as the maximum work efficiency from the experimental TMA results shown in Fig. 1d, where the heat capacitance of the coiled fibre was measured using a Thermo Plus DSC 8230 (Rigaku Corporation). The heat capacity values we used as an average value between 30 °C and 90 °C for LLDPE, HDPE and nylon6, 6 were 2 J g^−1^ K^−1^, 2 J g^−1^ K^−1^, and 1.5 J g^−1^ K^−1^, respectively. We also estimated η for Fig. 2c using the same heat capacities. In this case, the surface temperature of the fibres was measured via high-resolution infrared thermography (FSV-S550, Apiste Corporation). WAXD patterns were collected using a RINT-RAPID (Rigaku Corporation) operated at 40 kV and 30 mA via a ϕ300 μm collimator in transmission mode. SAXS patterns were collected using a three-slit XRD measurement system (Nano-Viewer equipped with a Pilatus K-100, Rigaku Corporation) in transmission mode. For observation of the fibre cross sections, we cut the fibres along the drawing axis using an ultramicrotome (Leica EM UC7, Leica Microsystems K. K.) at room temperature and measured the surface using SPM (SPA300HV + 3800N, SII Nano Technology Inc.) in tapping mode.

### Actuator fabrication

We fabricated two configurations of heater wires. We coated LLDPE coils with Ag conductive elastomer (PE872, DuPont Corporation) as follows: a couple of coiled LLDPE fibres twisted under 20 MPa of tensile stress were yarned into two-ply. Next, the twisted fibres were annealed under 10 MPa of tensile load for 20 min in an oven at 100 °C to eliminate any slack in the yarn. The surface of the yarn was then activated using an oxygen plasma treatment (Plasma cleaner, Harrick Plasma Inc.) and immediately brush-coated with PE872; it was then cured in an oven at 100 °C for 3 min (LLDPE/Ag-paste, Fig. 4c). 2-Ply yarn was necessary to keep the coils from untwisting. We also fabricated nylon6, 6 actuators using the same procedure, except the annealing temperature used to delete the slack was 200 °C (nylon6, 6/Ag-paste). In the second method, we yarned two coiled LLDPE fibres and Ag-plated nylon6, 6 multifilament(AgPoss, 100 denier, consists of 34 filaments, Mitsufuji Corporation) as a heater wire, which was twisted under a 98 mN load into stretchable coil form (Fig. 4a).

### Actuator characterization

We measured the displacement of fibre actuators using a laser displacement sensor (LK-080 sensor head attached to an LK-2000 amp. unit, Keyence Corporation). The temperature between the inner glove and finger skin was measured by inserting a micro thermocouple probe (SF-K-100-ANP, Anritsu Meter Co. LTD.). We measured the tension of the actuator required for bending the finger by attaching a spring balance to the end of the actuator.

### Glove fabrication

The LLDPE/heating-wire yarns were used to demonstrate bending of a finger by placing them on the index finger of a glove. We attached both ends of a bundle of three actuators onto metal snap buttons stitched onto the reverse side of Velcro fasteners. Input power was applied via these snap buttons. We stitched the middle of the actuators at the end of the finger of the fabric glove, placed them (six actuators in total) on the glove and then fixed them using Velcro in the expanded state to the back of the fabric glove, as shown in Fig. 5b.

### Analytical model of work efficiency

According to the model described in ref. 5, ∆L is not explicitly affected by tensile stress; the work efficiency of the coiled fibre actuator increases with increasing tensile stress. (For the expression of ∆L, see equation (S10) in the Supplementary information.) However, the tensile stress P/πd^2^, where P is the load applied to the coiled fibre, is limited by the breakdown shear stress:


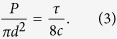


The work of coiled fibre actuator *A* is denoted as


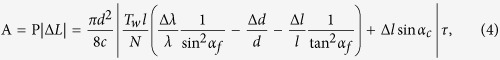


where α_c_ is fibre bias angle (See Fig. S4). A reaches a maximum at *τ* close to the breakdown shear stress. Moreover, the input energy *W* due to heating is denoted as


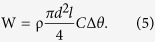


The expected work efficiency *η* is calculated from *η* = *A*/*W*; that is,


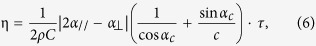


where we assumed 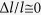.

## Additional Information

**How to cite this article**: Hiraoka, M. *et al*. Power-efficient low-temperature woven coiled fibre actuator for wearable applications. *Sci. Rep*. **6**, 36358; doi: 10.1038/srep36358 (2016).

**Publisher’s note:** Springer Nature remains neutral with regard to jurisdictional claims in published maps and institutional affiliations.

## Supplementary Material

Supplementary Information

Supplementary Movie S1
