# Supplementary material for: Power-efficient low-temperature woven coiled fibre actuator for wearable applications
Source: Sci Rep. 2016 Nov 4;6:36358. doi: 10.1038/srep36358 (PMC5095651; doi:10.1038/srep36358)
Supplement: Supplementary Information [file srep36358-s1.pdf]

# Supplementary information

# Power-efficient low-temperature woven coiled fibre actuator for wearable applications

**Maki Hiraoka*, Kunihiko Nakamura, Hidekazu Arase, Katsuhiko Asai, Yuriko Kaneko, Stephen W. John, Kenji Tagashira and Atsushi Omote**

## 1. Analytical model of work efficiency

As preparation for the derivation of work efficiency $\eta$, we first derive a formula between the displacement of coil length $\Delta L$ and deformation of the fibre surface caused by thermal strain of the fibre. This derivation is the same as that discussed in ref. [5], except that we reserve $\Delta l/l$ because it will be used to arrange the formula for $\eta$. Although the derived formula is considered only for the deformation of the fibre surface, we can roughly estimate how the thermal strain affects $\Delta L$ and $\eta$.

**1.1 Derivation of formula between $\Delta L$ and thermal expansion of fibre surface.** Fibre bias angle $\alpha_f$ is defined by considering the twisted angle of the fibre surface with diameter d, as indicated in Fig. S4:

$$\alpha_f = \sin^{-1}\frac{\pi n d}{\lambda} = \cos^{-1}\frac{l}{\lambda} = \tan^{-1}\frac{\pi n d}{l} \qquad \text{(S1)},$$

where $n$, $\lambda$, and $l$ are the number of twists around the fibre axis, the fibre length before twisting, and the fibre length after twisting, respectively. From the geometry shown in Fig. S3, λ is denoted as

$$\lambda^2 = l^2 + \pi^2 n^2 d^2 \qquad \text{(S2)}.$$

Here, we consider that the parameters of equation (S2) change slightly, which is denoted by adding Δ to the symbols of the parameters, i.e.,

$$(\lambda + \Delta\lambda)^2 = (l + \Delta l)^2 + \pi^2 (n + \Delta n)^2 (d + \Delta d)^2 \qquad \text{(S3)}.$$

By ignoring the second orders of tiny variations and substituting equation (S1) and (S2), we obtain a formula for $\Delta n/n$, which is also derived in ref. [5]:

$$\frac{\Delta n}{n} = \frac{\Delta\lambda}{\lambda}\frac{1}{\sin^2\alpha_f} - \frac{\Delta d}{d} - \frac{\Delta l}{l}\frac{1}{\tan^2\alpha_f} \qquad \text{(S4)}.$$

For comparison, equation (S4) can be converted to the formula denoted in ref. [5] by replacing $\alpha_f$ with $\pi/2 - \alpha_f$. When this twisted fibre is coiled, the total twist $T$ applied to the fibre is denoted as

$$T = n + N \sin\alpha_c \qquad \text{(S5)},$$

where

$$\alpha_c = \sin^{-1}\frac{L}{l} \qquad \text{(S6)}.$$

When rotation at the end of the twisted fibres is held to keep them from untwisting, $n$ and $N$ are assumed to be constant and the change in total twist is expressed using equations (S5) and (S6):

$$\frac{T+\Delta T}{l+\Delta l}-\frac{T}{l}=\frac{n}{l+\Delta l}-\frac{n}{l}+\frac{(L+\Delta L)N}{(l+\Delta l)^2}-\frac{LN}{l^2} \qquad \text{(S7)}.$$

We then arrange this equation for $\Delta L$ by ignoring the second orders of tiny changes and substituting equation (S6):

$$\Delta L=\frac{\Delta T l}{N}+\Delta l \sin\alpha_c \qquad \text{(S8)}.$$

Next, when the change in the number of twists of the fibre is assumed to be coupled with the change in the number of the twists of the coiled fibre, $\Delta T/T$ is denoted as

$$\frac{\Delta T}{T}=\frac{\Delta\lambda}{\lambda}\frac{1}{\sin^2\alpha_f}-\frac{\Delta d}{d}-\frac{\Delta l}{l}\frac{1}{\tan^2\alpha_f} \qquad \text{(S9)}.$$

From this equation, equation (S8) is derived as

$$\Delta L=\frac{Tl}{N}\left(\frac{\Delta\lambda}{\lambda}\frac{1}{\sin^2\alpha_f}-\frac{\Delta d}{d}-\frac{\Delta l}{l}\frac{1}{\tan^2\alpha_f}\right)+\Delta l\sin\alpha_c \qquad \text{(S10)},$$

where we assume that $\Delta l/l \cong 0$ and $\alpha_f = 45°$, as in [5]; $\Delta L$ is derived as

$$\Delta L=\frac{Tl}{N}\left(2\frac{\Delta\lambda}{\lambda}-\frac{\Delta d}{d}\right) \qquad \text{(S11)}.$$

This equation clarifies that negative thermal expansion of the fibre length $\Delta\lambda/\lambda$ and positive thermal expansion of the fibre diameter $\Delta d/d$ contribute to the large contraction of the coiled fibre actuator. The assumption of $\Delta l/l \cong 0$ implies that the deformation caused by thermal expansion is almost converted into the untwisting motion of the twisted fibre.

**1.2 Work efficiency of coiled fibre actuator.** Because equation (S10) is not explicitly affected by tensile stress, the work efficiency of the coiled fibre actuator increases with increasing tensile stress. However, the tensile stress $P/\pi d^2$, where $P$ is the load applied to the coiled fibre, is limited by the breakdown shear stress:

$$\frac{P}{\pi d^2}=\frac{\tau}{8c} \qquad \text{(S12)},$$

where $c$ is the spring index ($c = D/d$, where $D$ is the mean diameter of the coil). The work of coiled fibre actuator $A$ is defined as

$$\mathrm{A}=\mathrm{P}|\Delta L|=\frac{\pi d^2}{8c}\left|\frac{Tl}{N}\left(\frac{\Delta\lambda}{\lambda}\frac{1}{\sin^2\alpha_f}-\frac{\Delta d}{d}-\frac{\Delta l}{l}\frac{1}{\tan^2\alpha_f}\right)+\Delta l\sin\alpha_c\right|\tau \qquad \text{(S13)},$$

which reaches a maximum at a $\tau$ value close to the breakdown shear stress. Moreover, input energy $W$ due to heating is defined as

$$\mathrm{W}=\rho\frac{\pi d^2 l}{4}C\Delta\theta \qquad \text{(S14)}.$$

The expected work efficiency $\eta$ is calculated from $\eta = A/W$; that is,

$$\eta = \frac{1}{2\rho C}\left|2\alpha_{//} - \alpha_{\perp}\right|\left(\frac{1}{\cos\alpha_c} + \frac{\sin\alpha_c}{c}\right)\cdot\tau \qquad \text{(S15)},$$

where we assume $\Delta l/l \cong 0$.

Equation (S15) implies that the following factors contribute to high work efficiency: 1. Heat capacity, which reduces the heat capacity per volume of coiled fibre. 2. Thermal strain of fibre, which enlarges the anisotropic thermal expansion coefficient. 3. Shape of coiled fibre, which enlarges $\alpha_c$ and reduces $c$. 4. Stiffness, which enlarges the breakdown shear stress. The coiled LLDPE fibre inherently has the features of a large thermal expansion coefficient, small $c$ and large $\alpha_c$.

As discussed in the "Nanostructure analysis" section in the main text, these features originate from the nanostructure of the drawn LLDPE fibre, which contributes not only large thermal expansion but also affects the compact coil shape, thereby contributing to the high work efficiency.

For comparison, a general stiff coil spring with a compact shape, e.g., $c < 10$, breaks at $P$ values less than that described in equation (S12). The actual $\tau$ is known to be corrected to $\tau/\kappa$, where $\kappa$ is Wahl's empirical coefficient:

$$\kappa = \frac{4c-1}{4c-4} + \frac{1+2\nu}{2(1+\nu)c} \qquad (s16).$$

In this case, because $\kappa$ diverges when $c$ is reduced to 1, the breakdown shear stress decreases with reducing $c$. For example, the breakdown shear stress decreases by approximately half at $c = 2$ ($\kappa \approx 2$); the coils break at $c = 1$, even with no tensile stress applied. However, in the case of the coiled LLDPE fibre, whose diameter deforms into an ellipsoid because of applied stress, the shear stress applied to the inter-fibrils is relaxed; therefore, $\kappa$ does not work well even for small $c$ values. The apparent $c$ of the LLDPE shown in Fig. 1b is 0.7. We confirmed that such extreme deformation does not occur for hard crystalline polymers except for LLDPE, as listed in [5]. HDPE and nylon6,6 broke down at $c > 1$ when large tensile stress was applied during twisting to yield a coiled fibre with small $c$. At this point, deformation of the diameter of the LLDPE fibre contributes to the high work efficiency of the coiled LLDPE fibre actuator.

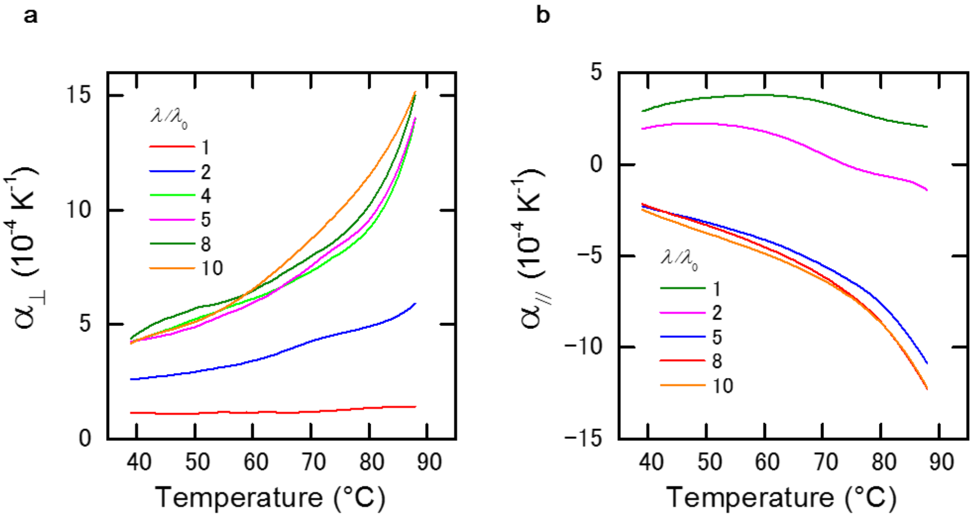


**Figure S1.** Thermal expansion coefficient of LLDPE fibres drawn in various drawing ratio $\lambda/\lambda_0$ a) along the polar direction ($\alpha_{\perp}$) and b) along the fibre axis ($\alpha_{//}$), as measured by TMA.

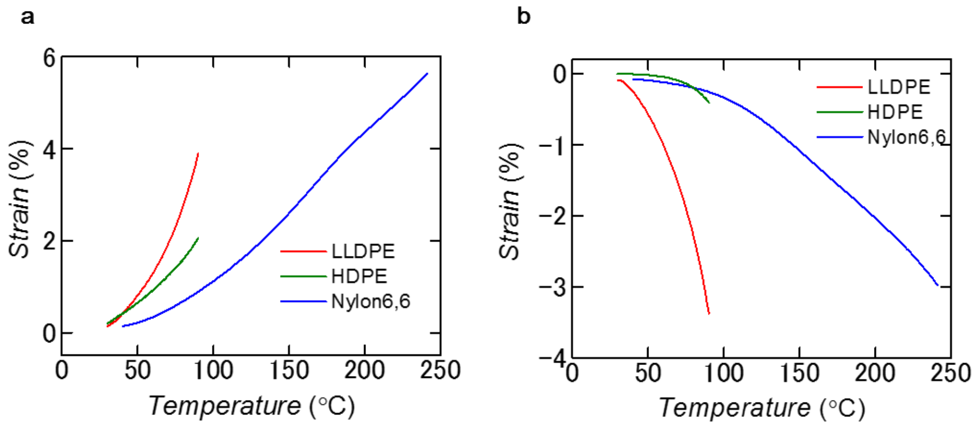


**Figure S2.** Drawn fibres fabricated from crystalline polymers. a) Thermal expansion of drawn fibres along the polar direction ($\alpha_{\perp}$) and b) along the fibre axis ($\alpha_{//}$), as measured by TMA.

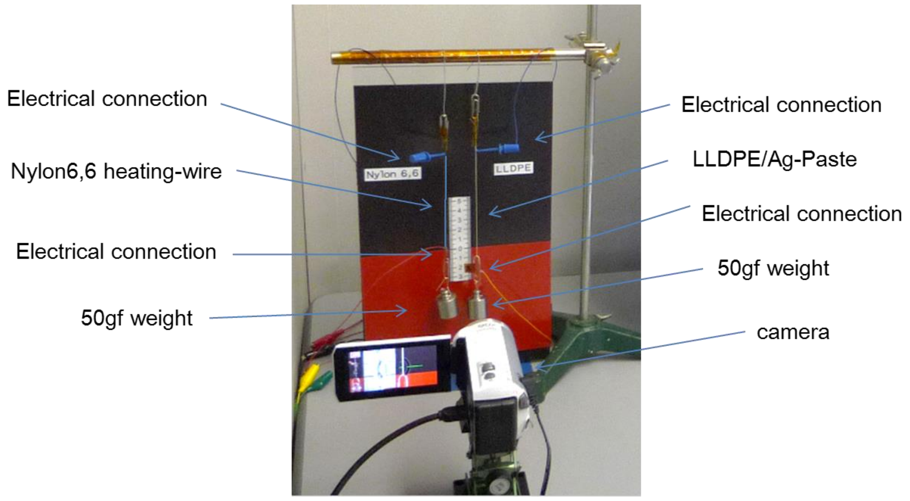


**Figure S3**. Experimental setup for MovieS1.mp4.

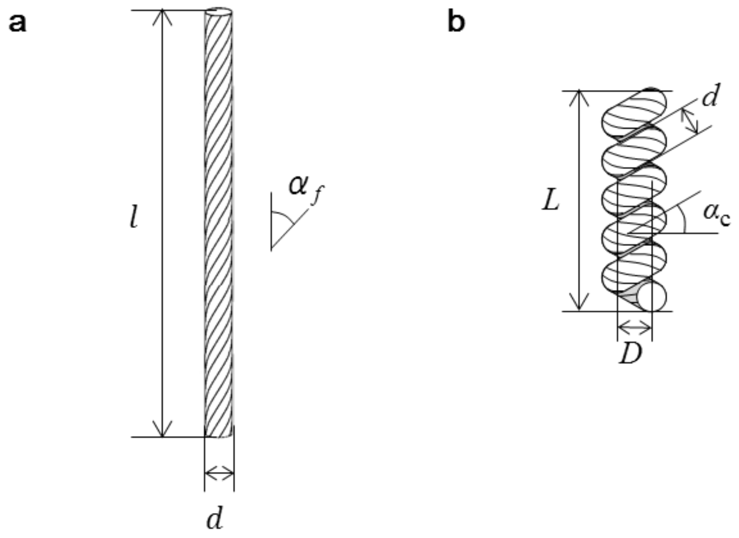


**Figure S4.** Schematics of a) a twisted fibre and b) a coiled fibre.

**Table S1.** Parameters used for estimation of actuation: conventional name, fibre diameter *d*, fibre length after twisting *l*, number of coiling *N*, number of twisting of fibre *T*, temperature reached by heating, liner expansion rate $\Delta\lambda/\lambda$, expansion rate along fibre diameter $\Delta d/d$, estimated actuation $\Delta L_{\mathrm{cal.}}/L$, and actual actuation $\Delta L_{\mathrm{exp.}}/L$ measured by TMA in Fig. 1d. The coil length is 10 mm.

| material | Parameters of twisting | | | | Heat expansion from 30°C | | | Actuation | |
|---|---|---|---|---|---|---|---|---|---|
| | $d$ | $l$ | $N$ | $T$ | *Temp.* | $\Delta\lambda/\lambda$ | $\Delta d/d$ | $\Delta L_{\mathrm{cal.}}/L$ | $\Delta L_{\mathrm{exp.}}/L$ |
| | mm | mm | turns | turns | °C | % | % | % | % |
| LLDPE | 0.12 | 24 | 61 | 89 | 49 | -0.55 | 0.8 | -6.7 | -5 |
| | | | | | 60 | -1 | 1.2 | -11 | -9 |
| | | | | | 90 | -3.5 | 3.9 | -39 | -23 |
| HDPE | 0.18 | 30 | 44 | 73 | 60 | -0.05 | 0.75 | -4.2 | -2.9 |
| | | | | | 76 | -0.15 | 1.3 | -8 | -5 |
| | | | | | 90 | -0.41 | 1.9 | -14 | -7.5 |
| PA66 | 0.13 | 29 | 50 | 76 | 60 | -0.12 | 0.33 | -2.5 | -1.8 |
| | | | | | 90 | -0.26 | 0.89 | -6.2 | -4.2 |
| | | | | | 100 | -0.3 | 1.1 | -7.5 | -5 |

**Table S2.** Parameters used for estimation of work efficiency: conventional name, density of fibre $\rho$, specific heat *C*, coil bias angle $\alpha_{\mathrm{c}}$, spring index *c*, operating temperature for actuation *T*, average linear thermal expansion coefficient $\alpha_{//\ \mathrm{ave.}}$ in the operating temperature, average thermal expansion coefficient along fibre diameter $\alpha_{\perp\ \mathrm{ave.}}$ in the operating temperature, tensile stress $\tau$, estimated work efficiency $\eta_{\mathrm{cal.}}$, actual work efficiency $\eta_{\mathrm{exp.}}$ measured by experimental, and Figures referred to take $\eta_{\mathrm{exp.}}$

| material | Parameters at r.t. | | | | Heat expansion coefficient | | | Load | Work efficiency | | |
|---|---|---|---|---|---|---|---|---|---|---|---|
| | $\rho$ | $C$ | $\alpha_c$ | $c$ | $T$ | $\alpha_{//\ \mathrm{ave.}}$ | $\alpha_{\perp\ \mathrm{ave.}}$ | $\tau$ | $\eta_{\mathrm{cal.}}$ | $\eta_{\mathrm{exp.}}$ | |
| | kg m$^{-3}$ | J g$^{-1}$ K$^{-1}$ | degree | | °C | $10^{-4}$ K$^{-1}$ | $10^{-4}$ K$^{-1}$ | MPa | % | % | source |
| LLDPE | 920 | 2 | 32 | 0.7 | 30-90 | -5.5 | 6.4 | 20 | 1.8 | 1.2 | Fig. 1d |
| | | | | | 25-55 | -2.5 | 3.7 | 20 | 0.9 | 1.1 | Fig. 2c |
| | | | | 0.5 | | | | 69 | 3.7 | 2 | Fig. 2c |
| HDPE | 960 | 2 | 20 | 1.1 | 30-90 | -0.6 | 2.7 | 20 | 0.27 | 0.3 | Fig. 1d |
| | | | | | 25-55 | -0.05 | 1.9 | 20 | 0.15 | 0.3 | Fig. 2c |
| | | | | | | | | 40 | 0.3 | 0.3 | Fig. 2c |
| nylon6,6 | 1140 | 1.5 | 15 | 1.2 | 30-90 | -0.4 | 1.4 | 20 | 0.16 | 0.2 | Fig. 1d |

**Movie S1.** Demonstration of the actuation. Twelve-centimetre-long coiled fibre actuators lifting 49 mN of weight. (left) Ag-plated coiled nylon6,6 multifilament (nylon6,6 heating-wire) and (right) 2-ply yarn of coiled LLDPE fibres coated with conductive elastomer (LLDPE/Ag-paste) are cyclically actuated by an input pulse of 2 W applied to each actuator for 1 sec after being in the power-off state for 6 sec. This movie was taken using the setup shown in Fig. S3 at room temperature (25 °C), with no air circulation.
